# Supplementary material for: Identification of Sesamin from Sesamum indicum as a Potent Antifungal Agent Using an Integrated in Silico and Biological Screening Platform
Source: Molecules. 2023 Jun 9;28(12):4658. doi: 10.3390/molecules28124658 (PMC10304600; doi:10.3390/molecules28124658)
Supplement: Supplementary file 1 [file molecules-28-04658-s001.zip › molecules-2428251-supplementary.pdf]

molecules

MDPI

Supplementary material

Identification of Sesamin from *Sesamum indicum* as a Potent Antifungal Agent Using an Integrated in Silico and Biological Screening Platform

Khushbu Wadhwa [1], Hardeep Kaur [1], Neha Kapoor [2,*] and Simone Brogi [3,*]

[1] Fungal Biology Laboratory, Ramjas College, University of Delhi, Delhi 110007, India
[2] Department of Chemistry, Hindu College, University of Delhi, Delhi 110007, India
[3] Department of Pharmacy, University of Pisa, Via Bonanno 6, 56126 Pisa, Italy
* Correspondence: nehakapoor@hindu.du.ac.in (N.K.); simone.brogi@unipi.it (S.B.);
Tel.: +91-011-27667184 (N.K.); +39-050-2219613 (S.B.)

Table of contents

Output of computational studies regarding the most promising hit compounds S2

Pinoresinol S2

Caflanone S4

Herbacetin S6

Table S1 S8

Table S2 S9

Output of computational studies regarding the most promising hit compounds

Pinoresinol

The docking output for pinoresinol is reported in Figure S1. The compound could interact with some crucial residues in the binding site such as Asp145, Asn146 and His254 by H-bonds, while it can establish strong hydrophobic interactions being able to form π-π stacking with Phe229 and Tyr255, while we detected a π-alkyl interaction with Phe144 and Phe258. Further interactions were detected with Glu192, Asn305, and Arg309. Moreover, pinoresinol is defined as the precursor of sesamin and due to the high structural similarity with sesamin, pinoresinol could target the Phe-Phe clamp through π-alkyl and carbon-hydrogen bonds at the +1 and +2 subsite of the enzyme (Figure S1). Based on these findings, pinoresinol could be able to interfere with the entry of β-1,3-glucan chain, inhibiting the morphogenetic switching.

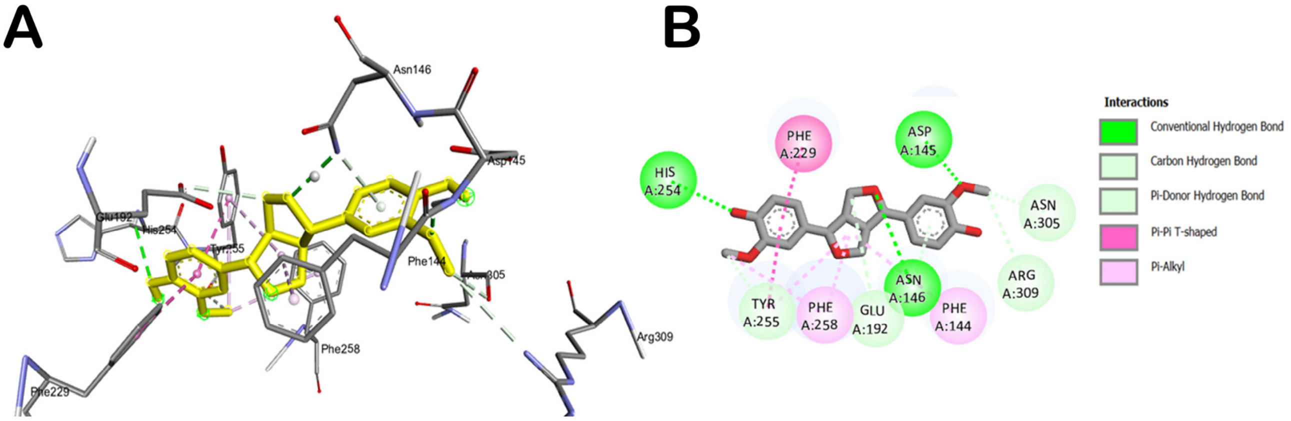

Figure S1. Main interactions of pinoresinol within the exo-1,3-β-glucanase binding site. (A) 3D model of interaction. (B) 2D schematic representation of the main interactions established by pinoresinol within the selected binding site of exo-1,3-β-glucanase.

To evaluate the behavior of pinoresinol within the binding site of exo-1,3-β-glucanase, we performed 100 ns MD simulation study on the complex found by docking studies, determining the stability of the complex and related time-line behavior. The results regarding different standard simulation parameters including RMSD and RMSF of each protein residue are reported in Figure S2. We observed global stability of the biological system, with no significant variations in structural stability observed upon binding of pinoresinol to the enzyme. Interestingly, the compound maintained the interactions found from molecular docking studies with no relevant movement within the binding site, preserving the interacting conformation (Figure S2A). The RMSF value represents the deviation between the protein atomic Cα coordinates and its average position throughout the MD run. The RMSF value is useful to determine the flexibility of specific protein backbone amino acids. As reported in Figure S2B, we detected a small fluctuation in the enzyme with the exclusion of some residues located at the C- and N-terminus sites.

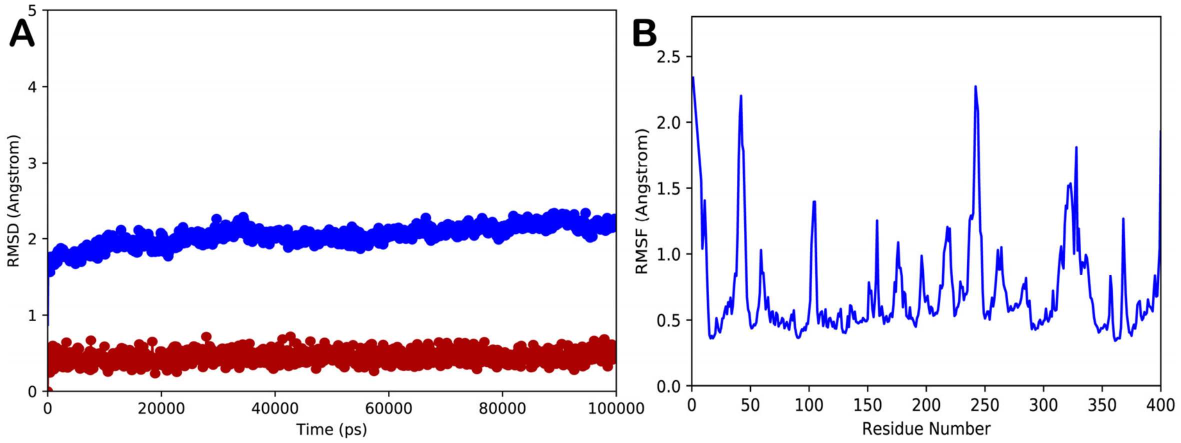

Figure S2. (A) Root-mean-square deviation (RMSD) regarding the protein/ligand complex (blue line for the protein and red line for the ligand); (B) Root-mean-square fluctuation (RMSF) of all residues of the protein. Pictures were created by Simulation Event Analysis available in Desmond.

To provide a complete overview regarding the dynamic behavior of pinoresinol within the selected binding site, we analyzed the main contacts formed by the ligand within the active site, reporting the time-line analysis of MD simulation (Figure S3, panel A and B). The H-bonds found by docking studies with Asp145 and Asn146 were maintained, while the H-bond with His254 was no longer detectable, being replaced by an H-bond with His253. The π-π stacking with Phe229 and Tyr255 were detectable during the whole simulation, as well as the contacts established by the ligand with Phe144, Glu192, Phe258, Asn305, and Arg309 (sometimes regarding Asn305, and Arg309 we observed water-mediated polar interactions). Overall, the computational outcome of the ligand within the selected binding site indicated that pinoresinol could produce stable interactions within the selected binding site, thus behaving as a potential ligand of the selected enzyme.

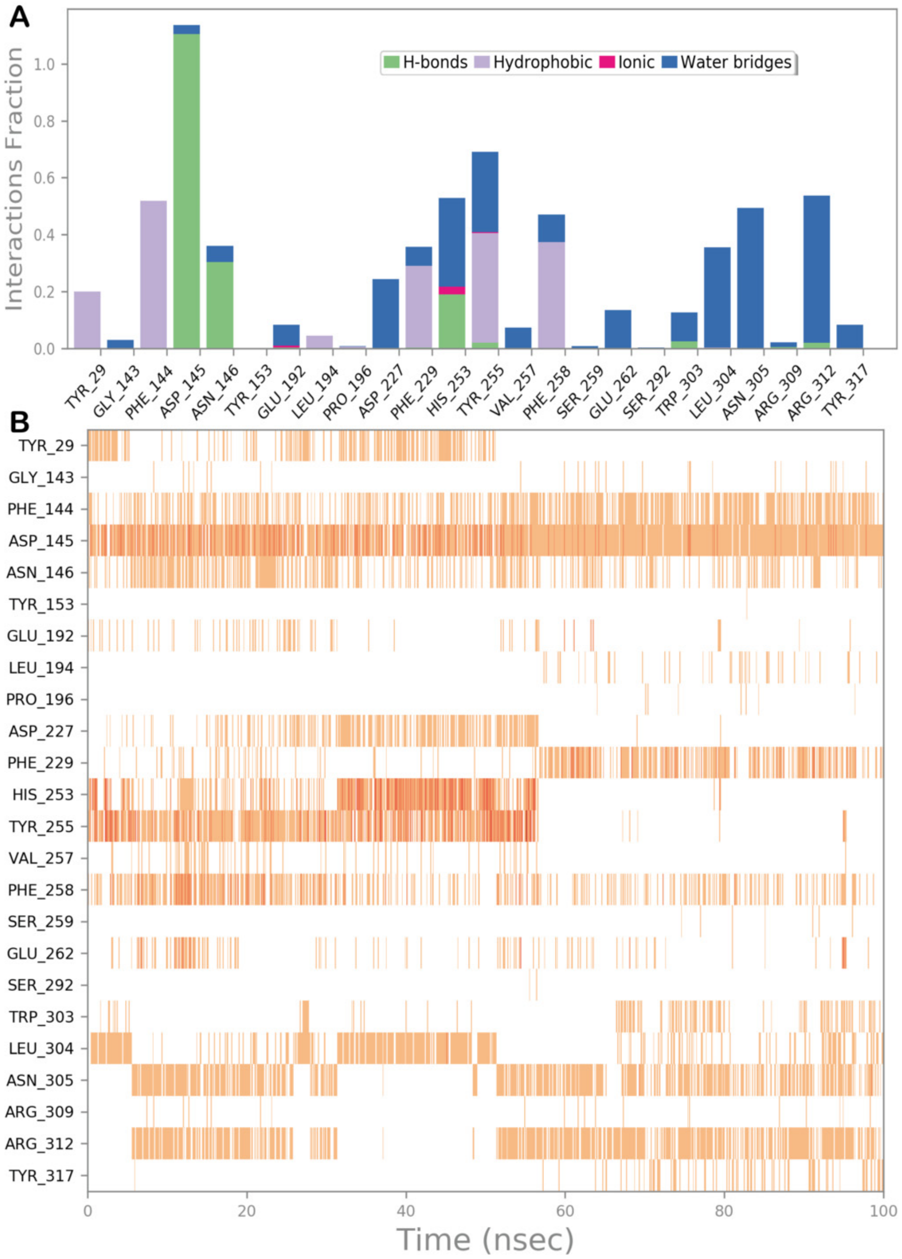

Figure S3. (A) Protein-ligand interactions monitored throughout the MD simulation. The interactions can be grouped into four types: H-bonds (green), hydrophobic (grey), ionic (magenta), and water bridges (blue). (B) The diagram

illustrates a timeline description of the main interactions. A darker hue of orange indicates that some residues make many distinct contacts with the ligand.

Caflanone

The docking output for caflanone is reported in Figure S4. The compound could interact with some crucial residues in the binding site such as Asp145 and Tyr255 by H-bonds, while it could establish strong hydrophobic interactions with His135, Phe144, Phe258, and Trp363. Considering the interactions found, the selected compound could inhibit the morphogenetic switching by interfering with the entry of β-1,3-glucan chain.

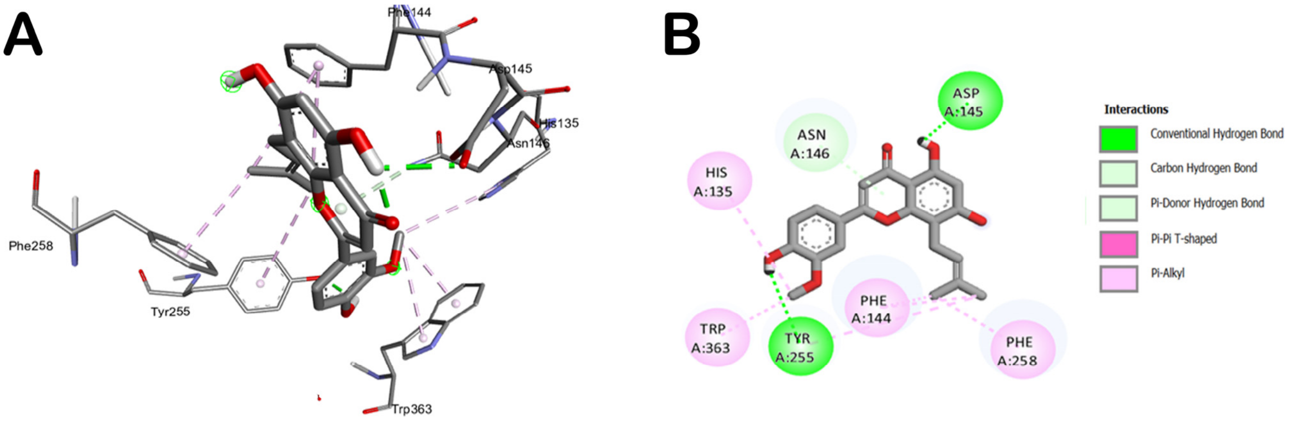

Figure S4. Main interactions of caflanone within the exo-1,3-β-glucanase binding site. (A) 3D model of interaction. (B) 2D schematic representation of the main interactions established by caflanone within the selected binding site of exo-1,3-β-glucanase.

The MD simulation results are reported in Figure S5. Considering the standard simulation parameters such as RMSD and RMSF of each protein residue, we observed global stability of the biological system, with no significant variations in structural stability observed upon binding of caflanone to the enzyme. Interestingly, the compound maintained the interactions found from molecular docking studies, with no relevant movement within the binding site, preserving the interacting conformation (Figure S5A). Regarding the RMSF value (Figure S5B), we detected a small fluctuation of the enzyme.

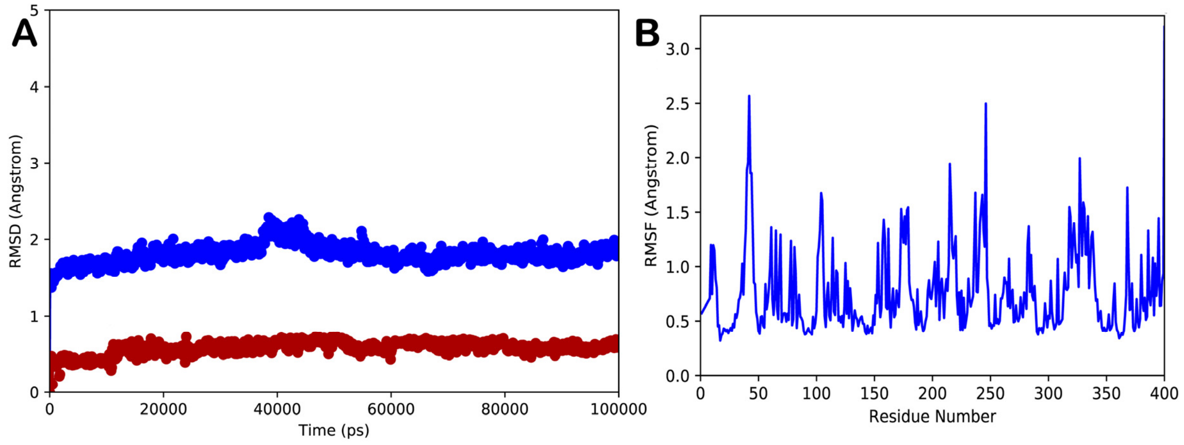

Figure S5. (A) Root-mean-square deviation (RMSD) regarding the protein/ligand complex (blue line for the protein and red line for the ligand); (B) Root-mean-square fluctuation (RMSF) of all residues of the protein. Pictures were created by Simulation Event Analysis available in Desmond.

The analysis regarding the dynamic behavior of caflanone along with its main interactions within the selected binding site is provided in Figure S6, panel A and B. The H-bonds found by docking studies with Asp145 and Tyr255 were still detectable during the simulation. In addition, caflanone was able to establish further polar contacts with Asn146, Arg312 and 317. The hydrophobic interactions with His135, Phe144, Phe258, and Trp363 were visible during the whole

simulation. In addition, hydrophobic interactions with Tyr29, Phe229 (part of Phe-Phe clamp), and Trp373 became more evident, and they could contribute to stabilize the retrieved binding mode. Overall, the MD investigation confirmed the capability of the ligand to establish fruitful contacts within the selected binding site, indicating that caflanone could strongly target the selected enzyme.

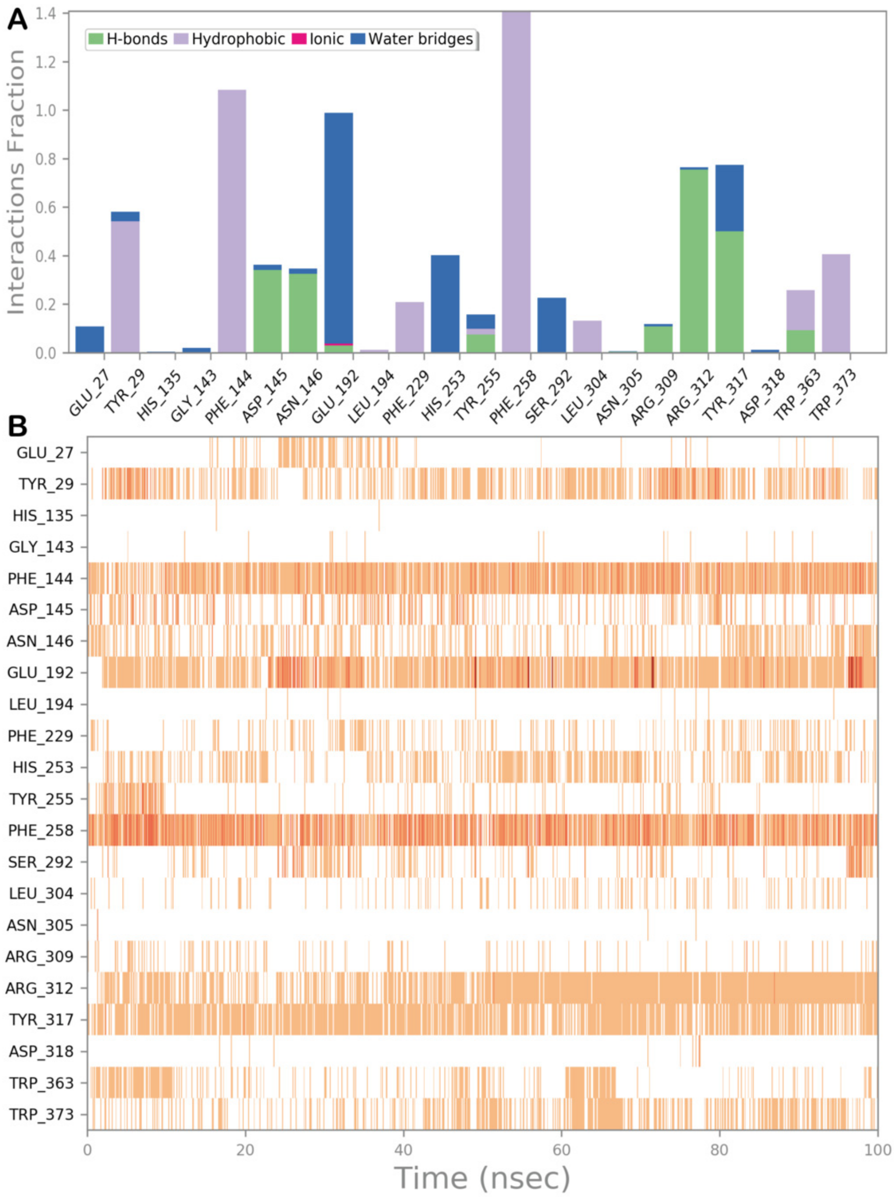

Figure S6. (A) Protein-ligand interactions monitored throughout the MD simulation. The interactions can be grouped into four types: H-bonds (green), hydrophobic (grey), ionic (magenta), and water bridges (blue). (B) The diagram illustrates a timeline description of the main interactions. A darker hue of orange indicates that some residues make many distinct contacts with the ligand.

Herbacetin

The docking output for herbacetin is shown in Figure S7. The compound could interact with some crucial residues in the binding site such as Glu27, Glu192, Ser292, Leu304, and Asn305 by H-bonds, while it can establish strong hydrophobic interactions being able to form π-π stacking with Phe258 and Trp363, while we detected further interactions with Asn146 and Tyr255. However, an unfavorable interaction was found with the residue Arg312. Accordingly, due to the pattern of interaction found, herbacetin could be capable of interfering with the entry of β-1,3-glucan chain and thus inhibiting the morphogenetic switching.

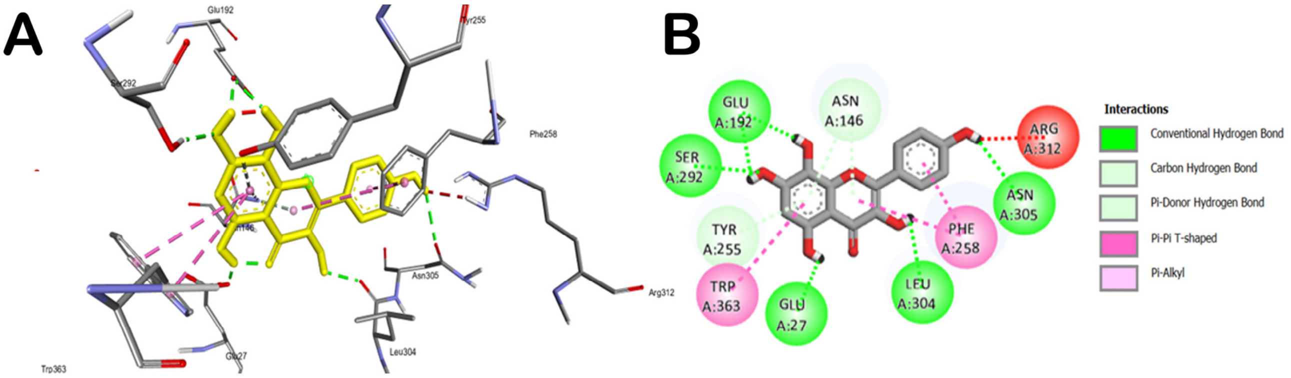

Figure S7. Main interactions of herbacetin within the exo-1,3-β-glucanase binding site. (A) 3D model of interaction. (B) 2D schematic representation of the main interactions established by herbacetin within the selected binding site of exo-1,3-β-glucanase.

The analysis of RMSD and RMSF, calculated from the MD trajectory, regarding the complex exo-1,3-β-glucanase/herbacetin is reported in Figure S8. Considering the mentioned parameters, we detected a general stability of the biological system, with no significant variations in structural stability observed upon binding of herbacetin to the enzyme. Interestingly, the compound maintained the interactions found from molecular docking studies, with no relevant movement within the binding site, preserving the interacting conformation (Figure S8A). Regarding the RMSF value (Figure S58B), we detected a minor fluctuation of the enzyme.

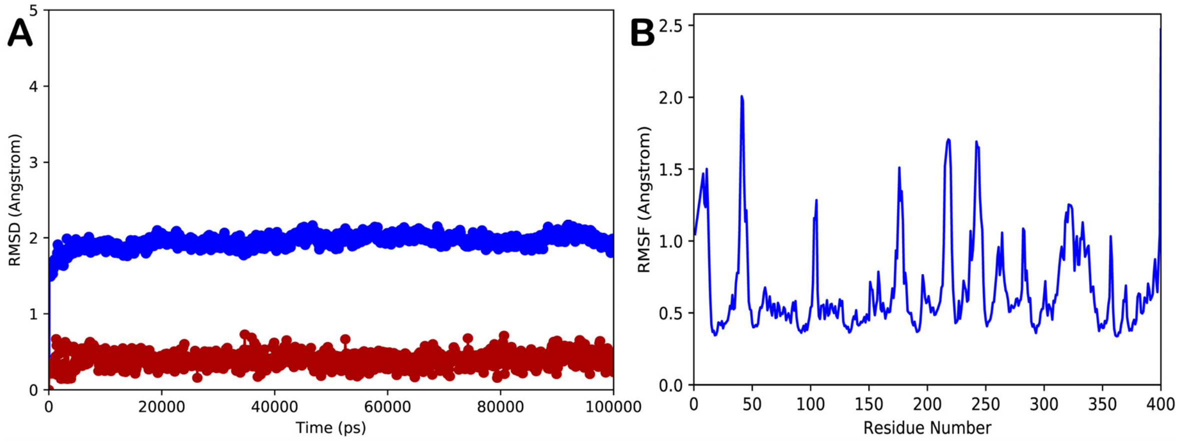

Figure S8. (A) Root-mean-square deviation (RMSD) regarding the protein/ligand complex (blue line for the protein and red line for the ligand); (B) Root-mean-square fluctuation (RMSF) of all residues of the protein. Pictures were created by Simulation Event Analysis available in Desmond.

Finally, the time-line contacts analysis of herbacetin within the selected binding site is reported in Figure S9 (panel A and B). The compound maintained the binding mode found by docking studies, but more favorable interactions were found with respect to those previously discussed. Briefly, the H-bond with Glu192 was evident, while the other H-bonds with Ser292, Leu304, Asn305, and Arg312 were found to be mainly water-mediated, and the contact with Glu27 became sporadic. The further contacts with Phe258 were strongly maintained, while those with Asn146, Tyr255 and Trp363 were detectable during the simulation. Interestingly, further π-π stacking with Phe144 and Phe229 were evident

and can contribute to stabilize the binding mode of herbacetin. Also in this case, the MD simulation confirmed the molecular docking output, indicating that the compound herbacetin could be a potential ligand of exo-1,3-β-glucanase.

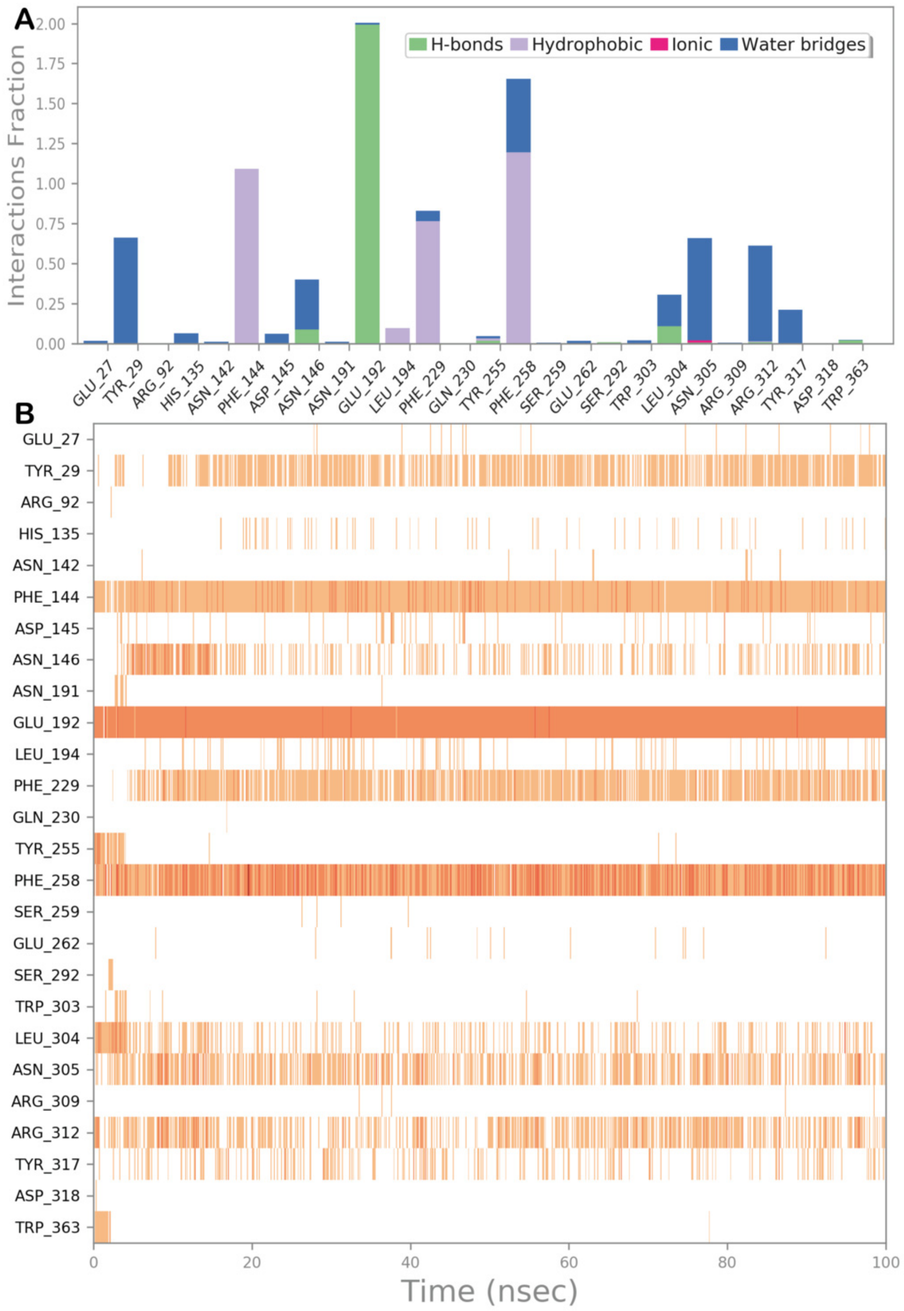

Figure S9. (A) Protein-ligand interactions monitored throughout the MD simulation. The interactions can be grouped into four types: H-bonds (green), hydrophobic (grey), ionic (magenta), and water bridges (blue). (B) The diagram illustrates a timeline description of the main interactions. A darker hue of orange indicates that some residues make many distinct contacts with the ligand.

In summary, the computational assessment highlighted that the top-ranking compounds could interact with the selected binding site of exo-1,3-β-glucanase due to the significant computational scores, but considering the behavior of the enzyme and its mechanism of action, we speculated about the different possibility of interaction since by observing the in silico results of sesamin, pinoresinol, caflanone, and herbacetin with exo-1,3-β-glucanase, only sesamin and its precursor were able to target both +1 and +2 subsite of enzyme, while the other compounds only marginally could target both sites.

Table S1. Screening of different classes of phytocompounds on the basis of the Rule of 5 and docking score (kcal/mol).

Serial number	Class	Phytocompound	Molecular weight	XlogP3	Hydrogen Bond Donor Count	Hydrogen Bond Acceptor Count	Docking Score (Kcal/mol)
(A)	Phenols						
1.		Acetophenone	120.15	1.6	0	1	-5.96
2.		4-Hydroxycinnamic acid	164.16	1.5	2	3	-7.86
3.		1,4-Benzoquinone	108.89	0.2	0	2	-6.64
4.		Caffeic acid	180.16	1.2	3	4	-8.82
5.		Vanillic acid	168.15	1.4	2	4	-8.66
(B)	Flavonoids						
6.		Caflanone	368.4	4.6	3	6	-11.48
7.		Herbacetin	302.23	2.2	5	7	-11.03
8.		Isoflavones	222.24	3.2	0	2	-8.05
9.		Baicalein	270.24	1.7	3	5	-9.82
10.		Luteolin	286.24	1.4	4	6	-10.42
(C)	Tannins						
11.		Gallic acid	170.12	0.7	4	5	-8.61
12.		Ellagic acid	302.19	1.1	4	8	-10.59
13.		Catechin	290.27	0.4	5	6	-10.79
14.		Fisetinidol	274.27	0.7	4	5	-10.59
15.		Robinetidinol	290.27	0.4	5	6	-10.77
(D)	Terpenoids						
16.		Geraniol	154.25	2.9	1	1	-6.62
17.		Carvacrol	150.22	3.1	1	1	-6.58
18.		Thymol	150.22	3.3	1	1	-6.55
19.		P-cymene	134.22	4.1	0	0	-5.52
20.		Citral	152.23	3	0	1	-6.39
(E)	Alkaloids						
21.		Protopine	353.4	2.8	0	6	-10.98
22.		Isoquinoline	129.16	2.1	0	1	-6.68
23.		Caseamine	327.4	2.6	2	5	-10.85
24.		Atropine	289.4	1.8	1	4	-9.53
25.		Quinine	324.4	2.9	1	4	-10.96
(F)	Quinones						
26.		5,8-Diamino-2,3-dimethyl-[1,4]Naphtoquinone	216.24	1.9	2	4	-8.74
27.		Dithymoquinone	328.4	2.1	0	4	-10.90
28.		Juglone	174.15	1.9	1	3	-7.46
29.		Thymohydroquinone	166.22	2.9	2	2	-7.30
30.		Thymoquinone	164.20	2	0	2	-7.26
(G)	Coumarins						
31.		Daphnetin	178.14	1.2	2	4	-8.66

32.		6-(3-carboxybut-2-Enyl)-7-Hydroxycoumarin	260.24	2.2	2	5	-10.97
33.		6-methoxymellein	280.21	2.4	1	4	-8.51
34.		Scopoletin	192.17	1.5	1	4	-8.25
35.		Umbelliferone	162.14	1.6	1	3	-7.81
(H)	Lignans						
36.		Pinoresinol	358.4	2.3	2	6	-11.50
37.		Matairesinol	358.4	3.3	2	6	-10.49
38.		Secoisolariciresinol	362.4	2.5	4	6	-10.44
39.		Sesamin	354.4	2.7	0	6	-12.21
40.		Magnolol	266.3	5	2	2	-8.66

Table S2. Various molecular docking poses of sesamin within the exo-1,3-β-glucanase binding site.

Conformer number	Binding energy (kcal/mol)	Inhibition constant (nM)	2D ligand-protein interaction of docked pose
11	-12.20	1.14	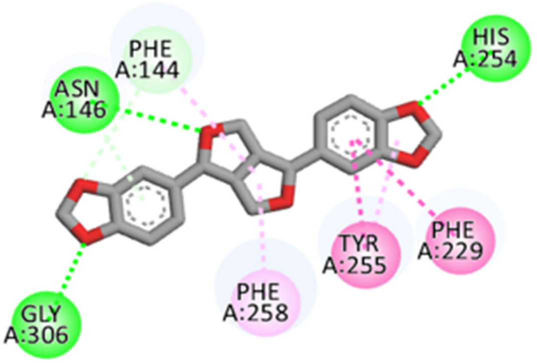
13	-12.19	1.15	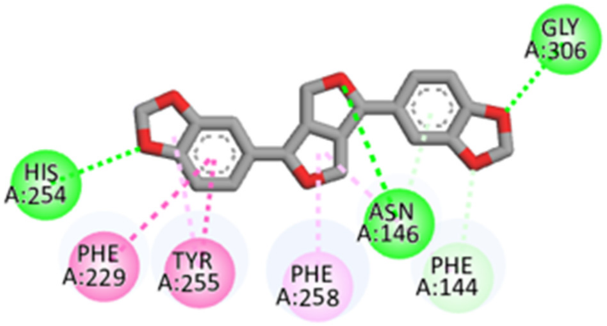

4	-12.18	1.17	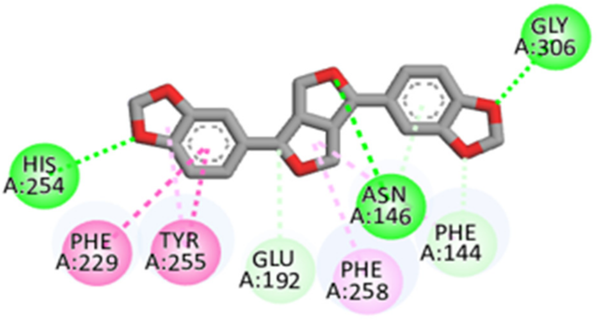
35	-12.17	1.19	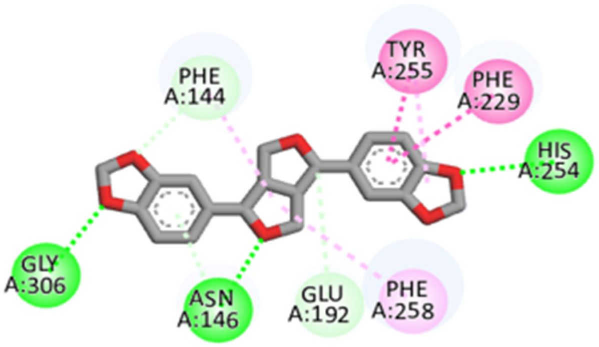
46	-12.16	1.21	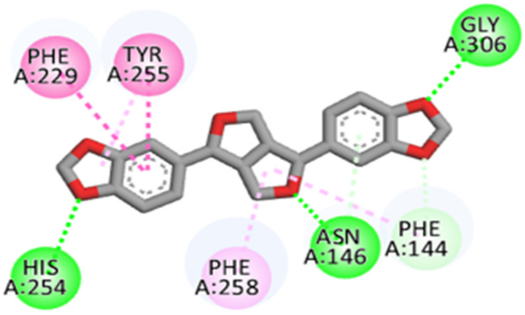
